# Supplementary material for: The Ubiquitin Ligase RNF138 Cooperates with CtIP to Stimulate Resection of Complex DNA Double-Strand Breaks in Human G1-Phase Cells
Source: Cells. 2022 Aug 17;11(16):2561. doi: 10.3390/cells11162561 (PMC9406464; doi:10.3390/cells11162561)
Supplement: Supplementary file 1 [file cells-11-02561-s001.zip › cells-1762478-supplementary.pdf]

Supplemental Figure S1

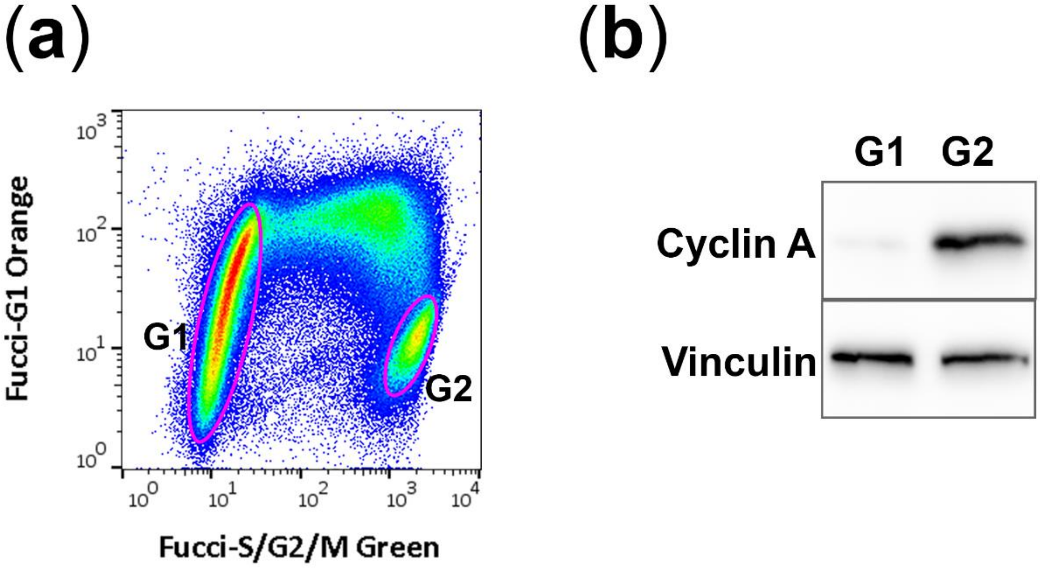

Supplemental Figure S1: Cell-cycle specific sorting of HeLa.S-Fucci. (a) Scatter plot of the cell-cycle specific fluorescence signals of Fucci-G1 Orange (G1 cells) and Fucci-S/G2/M Green (S, G2, and M cells). The gates to sort for G1 and G2 cells are highlighted. **(b)** Western blot analysis of cells sorted by flow cytometry for G1 and G2 cells. Whole cell extracts of sorted cells were immunoblotted for Cyclin A (S/G2 marker) and Vinculin (loading control).

Supplemental Figure S2

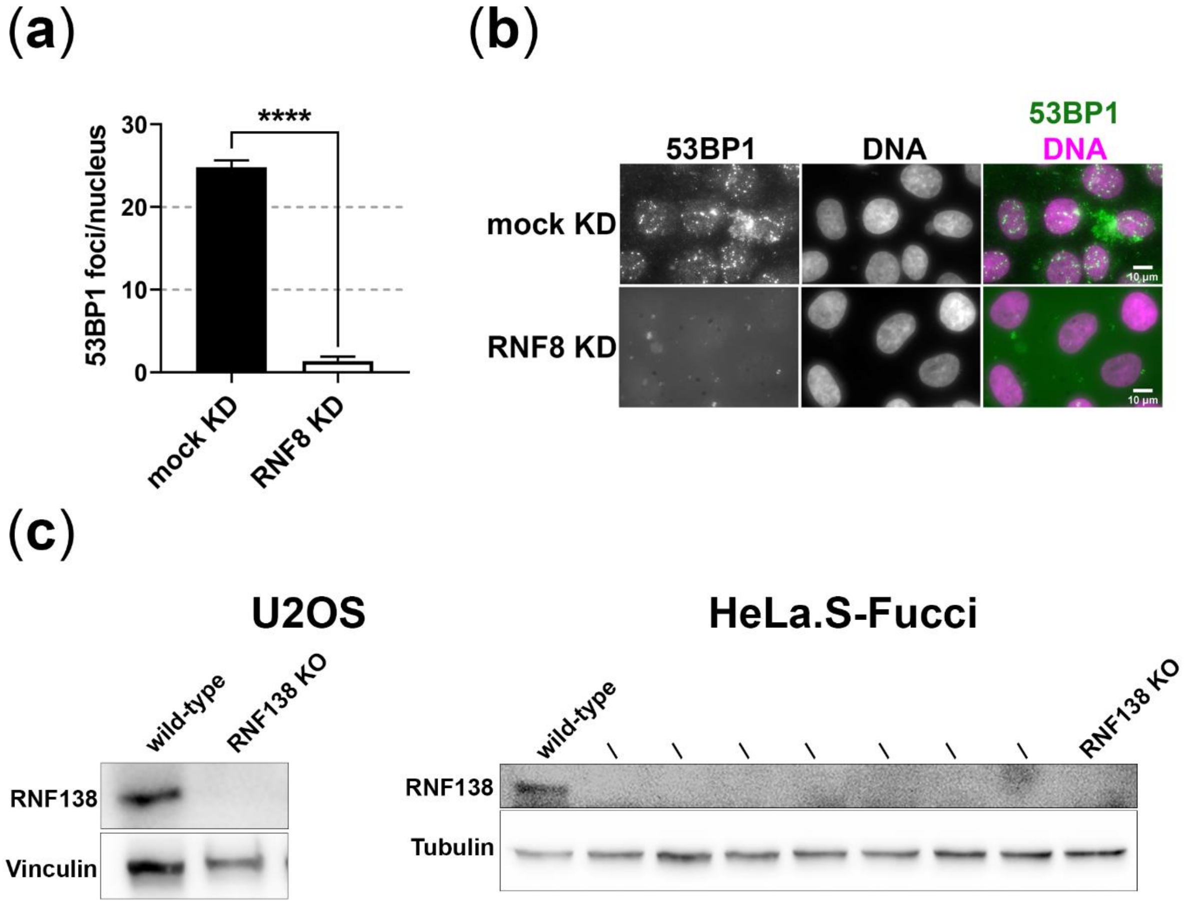

Supplemental Figure S2: Verification of RNF8 knockdown and RNF138 knockout. (a) Representative image of 53BP1 immunofluorescence stained cells irradiated with α-particles. DSB recruitment of 53BP1 requires histone ubiquitination by RNF8 and thus, quantification of radiation-induced 53BP1 foci can serve as a measure for an RNF8 knockdown (KD). U2OS cells were irradiated with 2 Gy α-particles and fixed 1 h after irradiation. 53BP1 was immunofluorescence stained and DNA was counterstained with DAPI. **(b)** 53BP1 foci were quantified and the foci number of non-irradiated cells was subtracted. Per condition, 100 cells were analyzed; n=1, error=SEM. Two-tailed Mann-Whitney-test, p values: **** $p < 0.0001$. **(c)** To verify the RNF138 knockout (KO) in U2OS and HeLa.S-Fucci cells, a western-blot analysis was performed, in which RNF138 and the loading control tubulin or vinculin was immuno-stained.

Supplemental Figure S3

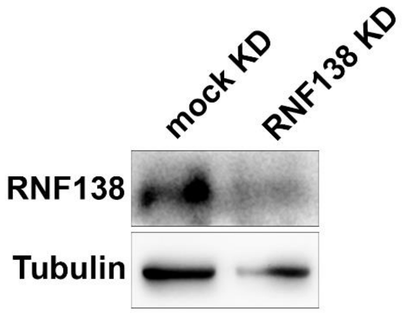

Supplemental Figure S3: Verification of RNF138 KD in NFFhTERT cells. Knockdown (KD) of RNF138 was verified by a western blot analysis. Tubulin served as loading controls.

Supplemental Figure S4

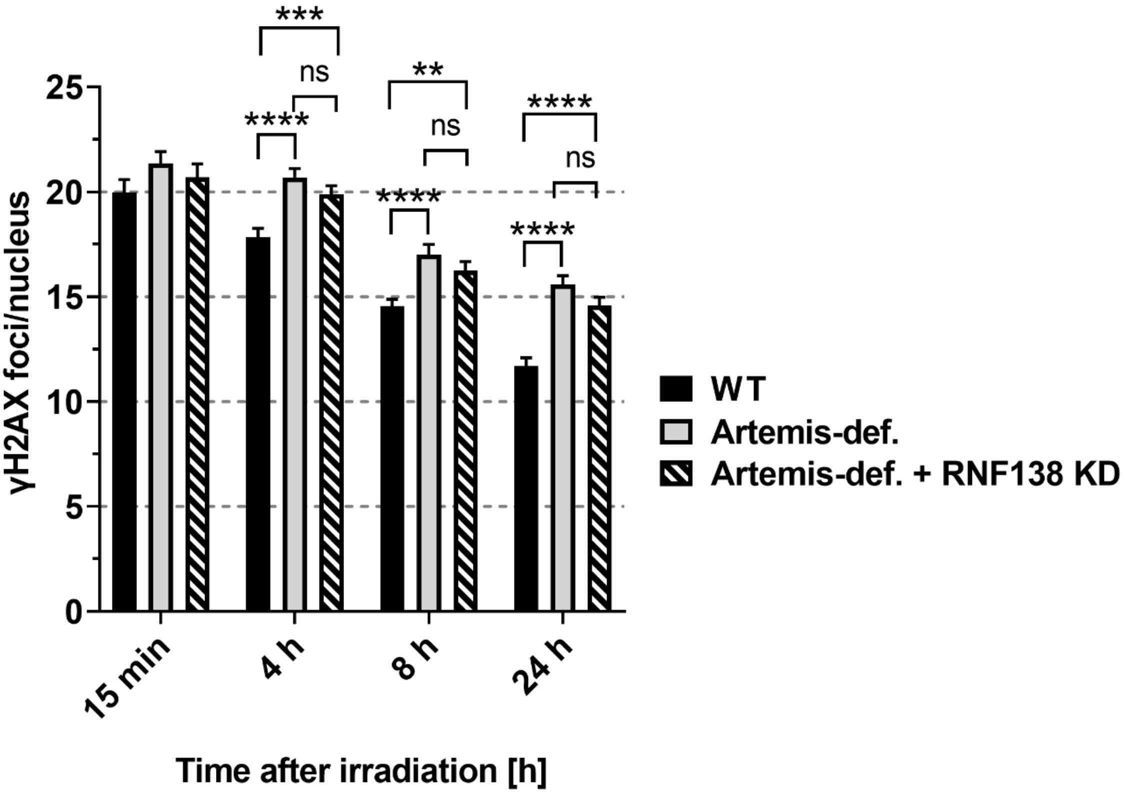

Supplemental Figure S4: At complex DSBs, RNF138 deficiency does not allow bypassing Artemis. Artemis proficient cells (82-6 hTERT) and deficient cells (CJ179hTERT) as well as Artemis deficient cells that were depleted for RNF138 by RNAi (CJ179hTERT + RNF138 KD) were irradiated with 2 Gy α-particles and fixed at different time points after irradiation. The samples were immunofluorescence stained for γH2AX and DNA was counterstained with DAPI. γH2AX foci/nucleus were quantified in at least 50 cells and averaged; n=1, error=SEM. Brackets indicate comparisons between samples. Two-tailed *t*-test, p values: ns, not significant; ** $p < 0.01$; *** $p < 0.001$; **** $p < 0.0001$.
